# Mechanisms and Biomarker Potential of Extracellular Vesicles in Stroke

**DOI:** 10.3390/biology11081231

**Published:** 2022-08-18

**Authors:** Nikita Ollen-Bittle, Austyn D. Roseborough, Wenxuan Wang, Jeng-liang D. Wu, Shawn N. Whitehead

**Affiliations:** 1 Vulnerable Brain Laboratory, Department of Anatomy and Cell Biology, Western University, London, ON N6A 5C1, Canada; 2 Department of Anatomy and Cell Biology, 454 Medical Sciences Building, The University of Western Ontario, London, ON N6A 3K7, Canada

**Keywords:** extracellular vesicles (EVs), cellular biology, inflammation, stroke, dementia, biomarkers, diseases

## Abstract

**Simple Summary:**

A stroke occurs when there is a lack of blood flow to the brain. Stroke injures the brain and can have devastating outcomes depending on the size and location of the brain tissue affected. Currently, there are only a limited number of treatment options for stroke. Extracellular vesicles are small vesicles secreted by cells. Importantly, extracellular vesicles have specific markers indicating the cell they were released from and can pass from the brain into the blood. For these reasons, assessing extracellular vesicles in the blood may create a window into changes occurring in the brain. Assessing changes in extracellular vesicles in the blood during stroke may produce new insight into the cellular changes in the brain causing injury during stroke. This in turn may generate potential targets for the development of future treatments. We summarize what is known about changes in brain-cell-specific extracellular vesicles during stroke and stress the importance of continuing to study these changes.

**Abstract:**

Stoke is a prevalent and devastating neurologic condition with limited options for therapeutic management. Since brain tissue is rarely accessible clinically, peripheral biomarkers for the central nervous system’s (CNS’s) cellular response to stroke may prove critical for increasing our understanding of stroke pathology and elucidating novel therapeutic targets. Extracellular vesicles (EVs) are cell-derived, membrane-enclosed vesicles secreted by all cell types within the CNS that can freely pass the blood-brain barrier (BBB) and contain unique markers and content linked to their cell of origin. These unique qualities make brain-derived EVs novel candidates for non-invasive blood-based biomarkers of both cell specificity and cell physiological state during the progression of stroke and recovery. While studies are continuously emerging that are assessing the therapeutic potential of EVs and profiling EV cargo, a vast minority of these studies link EV content to specific cell types. A better understanding of cell-specific EV release during the acute, subacute, and chronic stages of stroke is needed to further elucidate the cellular processes responsible for stroke pathophysiology. Herein, we outline what is known about EV release from distinct cell types of the CNS during stroke and the potential of these EVs as peripheral biomarkers for cellular function in the CNS during stroke.

## 1. Stroke

Stroke is major contributor to disability and is the second leading cause of death worldwide [1]. Acute ischemic stroke (AIS) comprises 87% of all strokes and occurs when there is partial or full obstruction of blood flow to a region of the brain, inhibiting the transfer of both oxygen and glucose to the tissue [2]. AIS can occur due to a global deprivation of blood flow as in after sudden cardiac arrest or as a focal insult following blockage of cerebral vasculature by thrombus or embolism. Following focal AIS, there is an ischemic core of irremediable damage where the blood flow has been cut off, but there is also a gradient of blood flow and respective tissue damage projecting out from the ischemic core. This is critical as the region surrounding the ischemic core, the penumbra, can potentially be rescued with timely restoration of perfusion [3]. Current treatments for AIS include administration of tissue plasminogen activator (tPA) to induce thrombolysis and endovascular thrombectomy to physically remove the blockage [4,5]. Transient ischemic attack (TIA) is a temporary perturbation in blood flow to the brain; however, unlike AIS, TIA typically resolves in a matter of minutes. Up to 12% of AIS cases are thought to be preceded by a TIA, suggesting TIA may indicate stroke risk [2].

Hemorrhagic strokes account for the remaining 13% of strokes and occur as a result of vascular rupture in the brain usually due to aneurysm or arteriovenous malformation [2]. Hemorrhagic stroke can be further divided based on the anatomical location of the bleed. Intraparenchymal hemorrhage (IPH) or intracerebral hemorrhage (ICH) refers to bleeding of non-traumatic origin into the brain parenchyma. Subarachnoid hemorrhage (SAH) refers to bleeding between the pia and the arachnoid membranes [6]. Hemorrhagic stroke presents significant risk as the increased pressure leads to mass effect and life-threatening events such as midline shift and brain herniation [7]. Hemorrhagic stroke is commonly treated with both pharmacologic and surgical therapies geared at controlling intercranial pressure and cerebral edema. Surgical interventions include aneurysmal clipping or endovascular coiling to prevent further bleeding and surgical decompression when appropriate [6]. Critically, hemorrhagic stroke must be differentiated from ischemic stroke to ensure proper therapeutic management. Ischemic and hemorrhagic stroke can be essentially indistinguishable symptomatically but require contrasting therapeutics. Critically, the pharmacologic management of each respective subtype can be detrimental to the alternate. The use of tPA for AIS runs the risk of generating or exacerbating a hemorrhagic stroke [8]. Additionally, while lowering blood pressure is used therapeutically for ICH, this management has been reported as potentially harmful in AIS patients [9,10,11]. Currently, differentiation between ischemic and hemorrhagic stroke is achieved through the use of medical imaging, including CT and MRI scans [12].

Characterizing the changes in cellular function that occur during stroke may not only help us better understand the distinct subtypes of stroke in both the acute and chronic stages but may elucidate novel targets for future treatments. More so than any other organ, brain tissue is rarely available to access clinically. For this reason, blood-based biomarkers offer unique potential in CNS injury to understand the processes or pathophysiologic state of the brain tissue. The ability to infer cellular activity in the brain in recovering stroke patients would produce novel insight into stroke pathophysiology and could be key to discovering targets for future treatments. 

## 2. The Need for Cell-Specific Biomarkers in Stroke

A variety of mechanisms exacerbate cellular injury post-stroke. Within minutes following the obstruction of blood flow to the brain, a drastic shift in ionic homeostasis causes spreading depolarizations (SDs) to occur [13,14,15]. SDs occur during stroke and recur over the following hours and days and radiate out from the initial region of injury into the surrounding area, irreversibly silencing neurons and spreading damage. In response to this depolarization, proximal cerebral blood vessels vasoconstrict [16,17]. This maladaptive coupling of vasoconstriction with depolarization further exacerbates ATP depletion and ultimately manifests as devastating disruption to the neurovascular unit (NVU). The NVU plays a critical role in brain homeostasis and comprises neurons, glia, and vascular and perivascular cells [18]. Following stroke, ionic perturbations, microglial activation, excitotoxicity, reactive oxygen species (ROS) production, and inflammatory mediators, including kinins, histamine, thrombin, substance P, endothelin-1, TNF-α, IL-6, and IL-1, have all been shown to contribute to the complex cascades leading to BBB dysfunction [19]. BBB dysfunction is a multifaceted process following AIS that subsequently leads to immune cell infiltration from the periphery, leakage of serum proteins, and perivascular inflammation [19,20]. The same holds true in hemorrhagic stroke; however, the BBB is disrupted in the zone of the hematoma as soon as the hemorrhage begins. Additionally, ICH is known to have different mediators of neuroinflammation, including thrombin and heme [21]. Collectively, cell-specific changes manifest a high degree of the pathophysiologic changes that occur during stroke in the hyperacute and acute timeframe. A way to peripherally detect cell-specific changes in the CNS may prove critical in generating novel therapeutic targets. 

The clinical outcome of stroke largely depends on the size and location of infarct, although sex and genetic factors can also play a role [22]. The National Institutes of Health Stroke Scale (NIHSS) is a scale used for the measurement of stroke severity and is highly correlated with clinical outcome [23]. Dynamic changes occur in the brain in the chronic stages post-stroke that encompass both re-mapping and neurodegenerative processes. Although only limited spontaneous functional recovery is seen in humans post-stroke, evidence from animal models suggests a window of neuroplasticity that could be capitalized on to promote re-training of neural networks [24]. In contrast, diaschisis or decreased neural functionality in a brain region distant from the infarct can occur as a result of persistent hypostimulation [25]. Although stroke is in part characterized by acute symptomology, these chronic changes can result in cognitive impairment and behavior changes even years later. Post-stroke dementia (PSD) is defined as any dementia that occurs post-stroke and may affect up to one-third of all stroke survivors [26]. At one year following a major stroke, the incidence of dementia is as much as 50 times higher compared to age- and sex-matched controls [27]. Stroke has been confirmed as a significant risk factor for all-cause dementia [28] and has been associated with chronic changes in white matter, medial temporal lobe atrophy, and Alzheimer’s disease (AD) pathology [29]. Additionally, stroke has also been corelated as a risk factor for the development of Parkinson’s disease (PD) [30]. Collectively, post-stroke changes in the brain increase the risk of future cognitive decline and neurodegenerative disease. The discovery of peripheral biomarkers that could reflect cellular function post-stroke would greatly increase our overall understanding of stroke pathology and could yield novel treatment targets. 

As previously discussed, under normal conditions, the brain is secured by the BBB. Although stroke results in disruption to this BBB, it holds true that the ideal biomarker is one that is freely able to pass through the BBB and elicit a picture of the physiologic state of the CNS. Additionally, the ideal biomarker would be of CNS origin and, moreover, would correlate with distinct cell types in order to offer a window into the physiologic processes occurring in the brain during stroke [31]. Such knowledge would not only improve our overall understanding of stroke but could also be critical in the discovery of future treatments. 

## 3. Extracellular Vesicles

The term extracellular vesicles (EVs) refers to a broad family of cell-derived, membrane-bound vesicles [32]. Secreted by all types of cells into extracellular fluids, EVs play important roles in waste removal and cell-to-cell communication. EVs display specific surface markers based on their cell of origin, which play a role in their intrinsic homing, and can be taken up by cells through various endocytic pathways (e.g., clathrin-dependent endocytosis, macropinocytosis) [32,33]. They are characterized according to their physical characteristics and biochemical composition, and can be divided into two major categories based on their route of formation: exosomes and microvesicles.

Exosomes, also known as intraluminal vessels, are the smallest category of EVs, ranging from 30–150 nm in diameter [32,34]. Exosomes are formed by an endosomal route from inward budding of early endosomes as they mature into multivesicular bodies (MVBs). MVBs are either sent to be degraded by lysosomes (whereupon the exosomes are degraded as well) or fuse with the plasma membrane to release its content, but the factors regarding the fate of a specific MVB are just starting to emerge [35]. Consequentially, the exact conditions for exosome production and release are also emerging as potentially clinically relevant processes. Studies have demonstrated that MVB fate may depend on cholesterol content in the endosomal membrane and regulation of the ESCRT pathway [36,37]. Exosomes contain a wide variety of different proteins and nucleic acids but generally include proteins associated with their endosomal secretion pathway (e.g., TSG101, HSC70) [32], higher levels of glycoproteins [32], and proteins involved in inter-organelle crosstalk [38].

**Figure 1:**
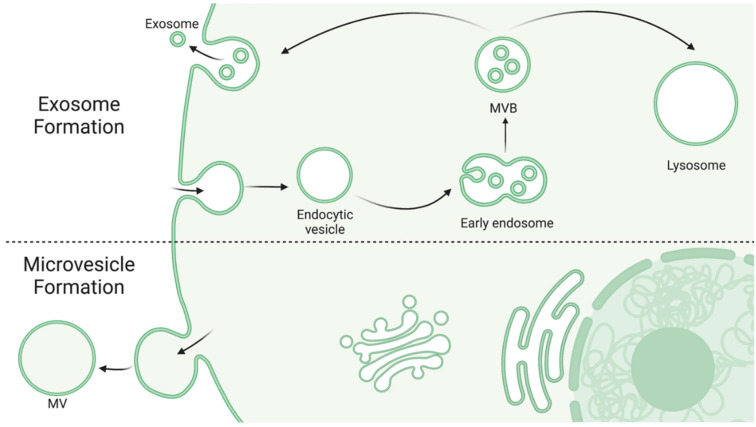
Exosome and MV formation. Exosomes are formed via the inward budding of early endosomes as they mature into MVBs [35]. MVBs are then either targeted to the lysosome for degradation, where as a result the exosomes are also degraded, or they fuse with the plasma membrane to release their contents [35]. MVs are larger in diameter and formed via the outward budding of the cell’s own membrane [32,33,34]. Created with BioRender.com.

Microvesicles (MVs), also known as ectosomes, are larger irregularly shaped vesicles, ranging from 50–1000 nm in diameter [32,34]. In contrast to exosomes, MVs are produced from direct outward budding of a cell’s plasma membrane. Figure 1 depicts the differential formation of exosomes and MVs. While knowledge of this route of formation is continuously emerging, it is thought to involve cytoskeleton restructuring and is highly dependent on the cell’s physiological state and microenvironment, perhaps in response to cell activation or injury [32,33,34]. Due to forming from the origin cell’s membrane, MVs generally display some of the cell’s surface markers as well and contain cytosolic proteins [34], heat shock proteins, and integrins [32]. Interestingly, glycan-binding proteins on the surface of MVs may be a key factor in understanding how MVs are targeted to and interact with other cells, suggesting that EVs released from specific cell types may show preference in uptake by other distinct cell types [33].

While both exosomes and MVs were originally thought to simply be a method of cellular waste removal, it has since been found that they play a key role in many biological processes. Their role in cell communication, regulation of microenvironments, and specificity to their parent and target cells have attracted significant scientific attention. EVs, particularly exosomes, have been studied extensively in the context of cancer for their potential role in metastasis, tumor microenvironment formation, and immune modulation [39,40,41,42]. Other studies have focused on vascular conditions such as cardiac fibrosis or diabetes for both diagnosis and treatment [43,44]. However, it is the ability of EVs to penetrate the blood-brain barrier (BBB) that has attracted the most recent attention, as they can be collected peripherally but used as a window into neuroinflammatory and neurodegenerative changes within the CNS [45]. EVs in the CNS have been shown to contain pathogenic proteins such as beta amyloid and alpha synuclein [32] and can propagate inflammation based on their parent cell’s activation state [33]. Herein, we will use EVs as a common term for all secreted vesicles as recommended by the International Society of Extracellular Vesicles (ISEV) guidelines [46].

## 4. Isolation and Characterization of Extracellular Vesicles

EVs’ small size and heterogeneous nature is the primary challenge for both isolation and characterization. In 2018, the International Society for Extracellular Vesicles provided updated guidelines on “Minimal information for studies of extracellular vesicles” (MISEV), which in summary follow these four guiding principles: (1) Describe the EV source using quantitative means; (2) demonstrate the presence of intact lipid bilayers and characterize purity; (3) utilize a combination of EV characterization techniques, preferably one optical and one biophysical/biochemical; and (4) when assessing the biological function of EVs, utilize a robust and rigorous experimental design [46,47]. 

The first step in this process typically involves the isolation of EVs from biological fluid. Common methods to achieve this include ultracentrifugation, precipitation, size exclusion chromatography, or density gradients. Tissue can also be used as an EV source. When isolating EVs from tissue, the additional step of tissue homogenization must be first carried out using dissociation buffers and physical shear stress to release the EVs trapped in the extracellular matrix [48,49]. Afterwards, debris can be pelleted out via centrifugation. It is important to note that the purity and overall EV yield varies between these methods; however, each is subject to the limitation of technical variability [50]. Further characterization of the EV subtype is also currently challenging due to a paucity of specific markers. For instance, CD9, CD63, and CD81 are thought of as classic exosome markers; however, they are also expressed by other EV populations [51]. Currently, density gradients with subsequent immunoprecipitation are a preferential method for the isolation of small, medium, and large EVs [51].

**Figure 2:**
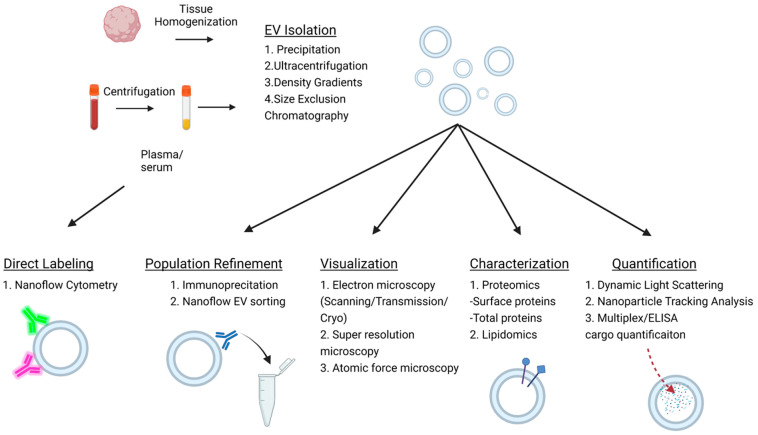
Methods of EV isolation and quantification. Schematic depicting commonly used methods for EV analysis. EVs can be measured in tissue homogenates or various biological fluids of interest (saliva, urine, blood, etc.). Plasma/serum isolation from blood is used here as an example. Using nanoflow cytometry, EVs can be directly labeled and quantified in fluids. Alternatively, EVs can be isolated and subjected to downstream analysis. Immunoprecipitation can be used to refine EV populations, EVs can be visualized using EM or other high-resolution microscopy approaches, and both lipidomics and proteomics can be used to characterize EV populations. Finally, quantification of EV concentration and size is possible with dynamic light scattering and nanoparticle tracking analysis while EV cargo can be quantified with sensitive protein or RNA assays. Created with BioRender.com.

Quantification is another important step in EV characterization. Many conventional approaches such as light microscopy and flow cytometry lack the requisite resolution for EV quantification. Newer technologies with higher resolution combating this limitation include scanning, transmission and cryo-electron microscopy, atomic force and super-resolution microscopy, dynamic light scattering (DLS), and nanoparticle tracking analysis (NTA). Figure 2 depicts the workflow of EV analysis and commonly used approaches for measurement of EV size, concentration, and cargo. Scanning, transmission, and cryo-electron microscopy can be used to image EVs and allow for size characterization and immunohistochemical labeling [52]. Atomic force and super-resolution microscopy have similar functionality; however, they are not able to detect smaller EVs [52]. DLS is a nanoparticle technology that measures light scatter due to Brownian movement to estimate particle size [53]. The primary limitation of DLS is the size results can be highly altered by the presence of large particles such as protein aggregate or dust within the sample [54]. NTA is a step up from DLS, utilizing the same principle of Brownian movement but detecting it in solution to detect size. NTA can be used to differentiate particle diameters between 30 and 1000 nm and generates a concentration measurement and visualization of the nanoparticles [54,55]. Moreover, NTA can be used in conjunction with fluorescent conjugated antibody labeling of the nanoparticles for specific subpopulation characterization [55]. 

Nanoflow cytometry represents a variation of flow cytometry that allows for multiparameter, high-throughput analysis of EVs and is optimized for the detection of EVs smaller than 1000 nm in diameter [56]. Nanoflow cytometry operates using a microfluidic system through which the nanoparticles pass, and multiple lasers of variable wavelengths generate light scatter, which is then measured by photodetectors, amplified, and is then translated to EV size and concentration measurement. Critically, since nanoflow cytometry does not require initial isolation and allows for detection directly from the biological fluid, it avoids the limitation of isolation variability [56]. In combination, multiple lasers and fluorophore-conjugated antibodies can be used to double or triple label EVs. Newer versions also provide sorting capabilities, which can further aid in the analysis of EV populations and their specific cargo.

EV cargo analysis also holds great interest for the development of EV biomarkers. Methods of EV cargo analysis include mass spectrometry, nucleic acid sequencing, sodium dodecyl-sulfate polyacrylamide gel electrophoresis (SDS-PAGE), Northern blots (for RNA), and Western blots (for protein) [47,48,49,51,57]. Each provides their own benefits and drawbacks based on the target species of interest. Additionally, proprietary technologies that integrate many of these techniques together in a more streamlined protocol, such as ExoView, have been developed to specialize in EV analysis [58,59]. Previous publications have reviewed EV isolation and characterization in detail [60,61,62].

## 5. CNS Cell-Specific Extracellular Vesicles as Biomarkers in Stroke

In addition to disruption of the BBB, the cellular changes and inflammatory mechanisms of injury following stroke also induce the release of EVs from the cells within the CNS and infiltrating cells from the periphery. EVs have been isolated from neurons, endothelial cells, microglia, astrocytes, oligodendrocytes, neural progenitor cells, erythrocytes, granulocytes, and leukocytes [35,63,64,65,66,67]. Importantly, EVs possess surface markers and cargo corresponding to their parent cell. Overall, the time course of BBB disruption, secondary injury, and related cell-specific EV release could offer insight into the pathophysiological changes in the brain following stroke. 

Collectively, EVs have the potential to serve as biomarkers indicative of changes in cellular function during stroke. EVs have been isolated from brain tissue but are also found in all biofluids, including cerebrospinal fluid (CSF), urine, and blood [68]. Common markers of brain-derived EVs include cell adhesion molecule L1 (L1CAM), glutamate receptors GluR2/3, GPI-anchored prion protein, neural cell adhesion molecule (NCAM), contactin-2/TAG 1, CD81, and other neuro-specific proteins, including neurofilament light (NF-L) chain, tubulin β, and neuron-specific enolase (NS-enolase) [69]. Additionally, miRNAs enriched within the CNS can be used, including miRNA-9, -124, -124a, -128, -135, -153, -185, and -219 [70]. Other more cell-specific markers will be discussed in the subsections below. Table 1 outlines the CNS cell types and their respective EV markers.

In addition to serving as biomarkers for changes in cellular function in the acute stages of stroke, EVs may also provide novel insight into changes occurring in the more chronic stages of stroke. For example, neuronal EVs have been suggested to transport proteins and miRNAs involved in synaptic plasticity while microglia-derived EVs were proven to stimulate synaptic regeneration and remyelination via membrane lipids [71]. The unique combination of EV surface markers and molecular cargo provides a plethora of accessible biomarkers for a comprehensive examination of brain health status post-stroke. The EV concentration can also provide information on the pathological state of the brain, as it was found to be increased during the inflammation process, correlated with neurological injury and diseases [72,73]. The mechanism underlying sustained EV changes may indicate a continued inflammatory response, brain edema, or impairment in the BBB [74]. As a result, interrogating the chronic elevation of EVs is critical to understanding the role of EVs in stroke resolution and the development of clinical sequelae.

Following acute ischemic stroke, the increased risk of cognitive impairment and dementia are major clinical concerns and require further investigation to understand their onset. Post-stroke EVs may play an integral role in damage resolution but may also create a neuronal environment susceptible to deterioration. Several publications have demonstrated the presence of pathological protein aggregates in EV cargo as disease-spreading vehicles in neurodegenerative diseases [75]. Thus, the increased propagation of misfolded proteins through EVs may be a mechanism promoting PSD. Interestingly, aggregated proteins associated with neurodegeneration can be detected as early as 1 h following acute ischemic injury and reperfusion, including the TAR-DNA-binding protein 43 associated with amyotrophic lateral sclerosis [76]. Several publications have also demonstrated the presence of beta-amyloid and tau released in association with exosomes [77,78,79] and neuronal cell-to-cell transmission of alpha-synuclein that contributes to neuronal death and cognitive impairment [75,80,81]. Together, EV secretion of pathological protein aggregates can propagate disease and exacerbate neurodegeneration in an already vulnerable brain post-stroke [82,83]. Herein, we summarize the current literature on EV release from cells of the CNS during stroke while highlighting their potential utility in serving as a peripheral window into changes in cellular function occurring in the CNS during stroke. We emphasize that future studies should consider cellular origin when assessing the potential of EV biomarkers in stroke.

**Table 1:** CNS cell types and EV markers. Table summarizing CNS cell types and their respective markers commonly used to assess EV cell origin.

CNS Cell	EV Marker	Reference Number
Endothelial Cells	Vascular endothelial (VE)-cadherin (CD144)	[84]
	Endoglin (CD105)	
	phosphatidylserine (PS)	
	Annexin V	
	Intercellular adhesion molecule 1 (ICAM-1) (CD54)	
	E-selectin (CD62E)	[85]
	Melanoma cell adhesion molecule (MCAM) (CD146)	[86]
	Angiopoietin-2	
	Collagen crosslinker lysyl oxidase-2	
Neurons	Synaptosome-associated protein 25 (SNAP25)	[87]
	Cell adhesion molecule L1 (L1CAM)	
	CD56	
	CD81	
Astrocytes	Excitatory amino acid transporter1 (EAAT1/GLAST)	[87]
	Glial fibrillary acidic protein (GFAP)	
Oligodendrocytes	Oligodendrocyte myelin glycoprotein (OMG)	[87]
	Myelin basic protein (MBP)	
Microglia	CD11b	[88]
	Transmembrane protein 119 (TMEM119)	
	CD13	[89]
	MCT-1	

### 5.1. Endothelial Cell-Derived Extracellular Vesicles as Biomarkers in Stroke

Following ischemic insult, endothelial cells undergo significant swelling and inflammatory stress, promoting the release of EVs [84,85]. Importantly, endothelial EVs can be further categorized by distinct phenotypic markers. Some express endothelial cell antigens such as vascular endothelial (VE), cadherin (CD144), or endoglin (CD105) [86]. Others are known to express phosphatidylserine (PS), a negatively charged phospholipid critical for the assembly of activation complexes requisite for coagulation that can be labeled with tag-conjugated annexin V, since annexin V is a protein with high affinity for PS [86]. An additional subpopulation of endothelial EVs express CD54, which may reflect a more inflammatory profile, as it is known that upon exposure to inflammatory cytokines, endothelial cells upregulate the expression of intercellular adhesion molecule 1 (ICAM-1) (CD54) [86]. Additionally, CD62E/E-selectin serves as a marker for activated endothelial cells and has been shown to be expressed on endothelial cells post activation by interleukin 1 (IL-1), TNFα, or bacterial lipopolysaccharides [90]. Other markers commonly used include CD146 or melanoma cell adhesion molecule (MCAM), which is highly expressed in endothelial cells and cell surface proteins such as angiopoietin-2 and the collagen crosslinker lysyl oxidase-2 [91].

Elevated levels of EVs post AIS were first reported in 2006 by Simak and colleagues, who identified an increase in endothelial EVs (PS^+^, CD105^+^, and CD41a^-^ (platelet marker)) relative to age-matched controls [85]. Moreover, they reported a correlation with stroke severity, with ICAM-1^+^ EVs (CD105^+^, CD54^+^, CD45^–^ (leukocyte marker)) being most strongly correlated with infarct volume and endoglin^+^ EVs (CD105^+^, CD41a^–^, CD45^–^) correlating with long-term clinical outcome. They demonstrated the ability to differentiate severe from minor AIS; however, they were not able to distinguish minor stroke from controls. A separate study assessing EV counts in TIA and AIS relative to healthy controls found a marked increase in CD146^+^, CD62E^+^, and annexin V^+^ EVs in both TIA and stroke within 48 h of the event, with a trend of stroke showing more endothelial EVs than TIA [92]. Similar increases were also observed in EVs with platelet, erythrocyte, granulocyte, and leukocyte origin within 48 h. At 5 and 30 days post-stroke, an increase in CD62E^+^ EVs in AIS but not TIA was noted. There were also increases in annexin V^+^ EVs along with EVs corresponding to platelets, granulocytes, and leukocytes but a decrease in erythrocyte-derived EVs in both AIS stroke and TIA. While this study did not confirm the cellular origin of annexin V^+^ EVs, a previous study that compared circulating EVs 48 h post AIS to high-cardiovascular-risk controls demonstrated an increase in annexin V^+^ EVs originating from endothelial cells (CD146^+^), neural progenitor cells (CD34^+^, CD56^+^), platelets (CD61^+^), erythrocytes (CD235ab^+^), and leucocytes (CD45^+^) [93]. In a more recent study examining plasma endothelial EVs (CD105^+^, CD144^+^, and annexin V^+^) specifically carrying miRNA-155, a marked increase was observed both in the acute (<24 h) and subacute (24 h to 2 weeks) stages post AIS and was positively correlated to NIHSS score and infarct volume [94]. Alternatively, brain endothelial cell EV miRNA-126 was found to decrease 3 h post-ischemia in both transient and permanent ischemia conditions in rats and returned to baseline at 24 h. Intriguingly, total serum miRNA-126 levels appeared to only decrease in permanent ischemic conditions, suggesting serum miRNA-126 may be able to serve as a biomarker for stroke severity [95]. However, the future benefit of EV analysis is the accrual of knowledge with respect to the cell specificity of miRNA-126.

As previously described, AIS and ICH are known to have different mechanisms of cellular injury. Given the relationship between endothelial cells and vascular integrity, it stands to reason EVs released from endothelial cells may provide unique insights into ischemic vs. hemorrhagic insult in the brain. Previous studies have demonstrated an increase in annexin V^+^ EVs in ICH patients compared to controls [64,96,97], and even more specifically have demonstrated an increase in endothelial EVs (CD105^+^, CD106^+^, CD54^+^, or CD62E^+^) along with EVs of other origins [98]; this may not be specific as an increase in endothelial EV release is observed in AIS as well. Although, at this time, endothelial EVs do not differentiate stroke subtypes, it is possible this could change, with further studies emerging on endothelial EV cargo variations. For example, the direct contact of blood components with the brain parenchyma in IHC versus the hypoxic environment of AIC may drive unique miRNA signatures reflected by EV release. 

EVs have also been shown to demonstrate potential as indicators of cerebral small vessel disease, suggesting a utility for them in the detection of ongoing endothelial dysfunction occurring in the post-stroke scenario [99]. Considering the damage occurring to the BBB following AIS, it is probable that EVs derived from endothelial cells provide unique insight into BBB integrity. Specifically, endothelial cells are known to play a critical role in promoting synergy between neurogenesis and angiogenesis during brain repair [71]. Sustained EV changes following the hyperacute phase of ischemic stroke can be reflective of ischemic severity that suggests worse clinical prognosis and recovery. EVs released from endothelial cells show change in their phenotypic profile that correlates to increased ischemic stroke risk and damage [100]. Additionally, greater CD62E^+^ endothelial EVs are significantly associated with recent ischemic episodes, increased NIHSS scores, and infarct volumes in acute stroke patients [100].

Beyond stroke severity, endothelial EVs have been explored as biomarkers to monitor treatment efficacy following acute ischemic stroke. In a case-control study, E-selectin, P-selectin, and platelet-derived EVs were all significantly higher within 7 days of stroke onset. At 3 to 6 months after stroke, E-selectin and P-selectin fell significantly below controls while platelet-derived EVs remained elevated to reflect sustained platelet activation in the recovery phase [101]. In conclusion, EVs derived from endothelial cells reflect the ischemic severity and vascular damage as indicators of long-term clinical outcome and may serve as promising biomarkers to monitor therapeutic efficacy post-stroke. In addition to their role as potential biomarkers, increasing evidence is emerging of neuroprotective effects of endothelial-derived EVs [102]. While this is outside the scope of this article, it is possible endothelial-derived EVs offer a novel therapeutic approach to stroke.

### 5.2. Neuron-Derived Extracellular Vesicles as Biomarkers in Stroke

Ionic perturbations, ATP depletion, SD, and other secondary mechanisms of injury, including excitotoxicity, ROS production, mitochondrial failure, microglial activation, pro-inflammatory cytokine release, and infiltrating immune cells from the periphery, all impact neuronal wellbeing [19]. How neuronal EVs reflect the local tissue changes post-stroke is not fully understood; however, it is likely they influence post-stroke pathophysiology given their known role as a mediators of intercellular communication [87]. Synaptosome-associated protein 25 (SNAP25), L1CAM, CD56, and CD81 are markers that are enriched in EVs of neuronal origin [103]. 

Interestingly, a recent study proposed that neurons release EVs containing miRNA-98 as a “help me” signal during stroke, which in turn causes a decrease in microglial phagocytosis of stressed but still viable neurons in the penumbral region. Importantly, in rats subjected to MCAO, miRNA-98 expression was found to peak in the penumbra on the first day but rapidly declined 3 days post-stroke [104]. Separately, neurons have been found to release EVs containing miRNA-124a, which results in an increase in glutamate transporter 1 expression and increased glutamate uptake by astrocytes [105]. Taken together with the fact that miRNA-124 increases post-stroke, it is possible this occurs in stroke as a response to excitotoxic changes. Another recent study examined the miRNA content of EVs from rat cortical primary neuronal cell cultures in oxygen glucose deprivation (OGD) vs. normoxic conditions and found the expression of 45 EV-contained miRNAs was significantly altered [106]. While separate studies have reported the potential beneficial effects of neuron-derived EV cargo, including miRNA-181c-3p, it remains to be determined if the release of this cargo is timed with the acute stage of stroke [107]. 

A recent study found several EV miRNAs demonstrating differential expression in AIS vs. ICH. One of these miRNAs was miR-134, which has been predominantly found to be a neuronal miRNA. Critically, this study did not include a non-stroke control group, making true assessment of the diagnostic potential of these miRNA challenging [108]. Interestingly, a recent study comparing subcortical (SC) and cortical-subcortical (CSC) AIS found specific expression of neuron EV-contained proteins C1QA and Casp14 in the CSC group and ANXA2 in the SC group [109].

Dynamic neuronal EV changes are also associated with functional rehabilitation in patients with subacute stroke. Platelet-derived EVs were found to form the largest population post-stroke, followed by neuronal and endothelial-derived EVs [110]. Critically, low neuronal and platelet EVs at baseline were associated with poor activities of daily living, whereas a slight increase in neuronal EVs was associated with improved prognosis in the first 6 months post-stroke [110]. Chronic neuronal EV changes may therefore provide novel insight into cellular post-stroke changes occurring in the brain, driving injurious and reparative cellular processes. 

### 5.3. Microglia-Derived Extracellular Vesicles as Biomarkers in Stroke

Microglia are the resident immune cell of the CNS and play a complex role in stroke pathophysiology. Neuroinflammation stems from the activation of microglia within the brain and infiltration of leukocytes from the periphery due to BBB dysfunction [19,111]. Following neural injury from stroke, microglia respond to damage-associated molecular patterns (DAMPs) released from injured cells [112]. Generally speaking, microglial activation involves retraction of their ramifications to take on an ameboid shape with phagocytic properties [113]. While activated microglia can be thought of as having either a pro-inflammatory or anti-inflammatory phenotype, this is now thought to be an oversimplified understanding and it is probable that microglial activation exists along a spectrum of these phenotypes [114]. Microglial response to neural injury is a rapid and dynamic process. Upon laser ablation or excess ATP exposure mimicking dying neurons, microglia extend their ramifications and envelop the site of insult within minutes [115]. Perhaps even more shockingly, a recent study using immunohistochemical analysis of post-mortem traumatic brain injury brains detected activated microglia and phagocytosed erythrocytes within the contusion zone even in those with just minute-long survival post injury [116]. Complex spatiotemporal relationships fuel microglial polarization in the subsequent hours, months, and even years post-stroke. Evidence suggests a shift to a more proinflammatory microglial polarization in the chronic stages [117,118]. While in the acute phase, microglia may play a protective role, as selective eradication of microglia in rats exacerbates infarct volume following middle cerebral artery occlusion (MCAO) [88]. Collectively, although AIS and ICH are associated with increased pro-inflammatory microglial responses, microglia are seemingly also involved in reparative processes in both the acute and chronic stages.

The microglia-macrophage-specific protein CD11b is commonly used as a marker for microglial-derived EVs, and the more specific microglia transmembrane protein 119 (TMEM119) is also expressed on microglial EVs [89]. CD13 and MCT-1 are additional markers that are highly enriched, but not specific to, microglial EVs [119]. In keeping with their role in immune function, microglial EVs bear membrane receptors and contain cargo similar to dendritic cells and B lymphocytes, including enzymes such as chaperones and tetraspanins [120]. Microglia have also been found to release different EVs depending on their environment and activation state. A study examining BV2 microglial-derived EVs found cells stimulated with LPS released more EVs with greater levels of pro-inflammatory cytokines, including IL-6 and TNFα [72]. It is not only the cargo of EVs that is affected, as exposure to ATP alters the proteome of microglial EVs [121]. A more recent paper investigated the EV content of microglia pre-conditioned with OGD solution to simulate stroke. Interestingly, the EVs derived from these microglia were found to be abundant in TGF-β1. Treatment of OGD-stressed primary cortical neurons and endothelial cells with these EVs attenuated neuronal injury and promoted angiogenesis, respectively. Moreover, exposure of cultivated microglia to these EVs resulted in an increase in anti-inflammatory-type polarization (CD206^+^/Iba1^+^) [122]. Taken together, it is apparent that microglia may respond uniquely to different environmental stressors, which in turn may drastically alter their respective EV profiles. In keeping with the anti-inflammatory profile acutely following ischemic insult, a separate study found an increase in miRNA-124 in anti-inflammatory-type microglial EVs and found miRNA-124 was upregulated in the ischemic penumbra 72 h following transient MCAO [123]. Moreover, anti-inflammatory microglia-derived EVs were found to be neuroprotective via miRNA-124 following MCAO in the mouse brain [123]. In contrast, EVs from OGD-activated microglia have been shown to damage endothelial cells through EV miR-424-5p modulation of the fibroblast growth factor (FGF2)/STAT3 pathway [124]. 

It is clear that microglia play a dynamic role in both injurious and neuroprotective post-stroke processes. As reviewed by Taylor and Sansing, several studies taken together outline the timeline by which anti-inflammatory-type microglia initially predominate the ischemic core, followed by a shift to pro-inflammatory-type microglia over the course of 2 weeks [21]. Interestingly, following ICH, phenotypic pro-inflammatory markers increase and peak as soon as 4 h following the event, and are then shown to decrease at 7 days [125]. Moreover, ICH is known to have different mediators of microglial activation, including thrombin and heme [21]. As previously noted, EV profiles differ between LPS-stimulated and OGD-stressed microglia. It is possible the EV profile is also different between ischemic and hemorrhagic insult. In keeping with this idea, since microglial activation exists along a highly complex spectrum from anti-inflammatory to inflammatory that is affected by mediators of activation, single-cell transcriptomics studies may be used as a template to correlate the microglial activation state with EV cargo and subsequently allow for phenotype-specific biomarker discovery. Collectively, further characterization is needed of microglia-specific EVs and their cargo during the hyperacute, acute, and chronic stages of stroke, but it is probable that microglial EVs could offer insight into activation patterns of microglia post-stroke.

### 5.4. Astrocyte-Derived Extracellular Vesicles as Biomarkers in Stroke

Astrocytes are the most abundant cell within the CNS and play critical roles in BBB permeability and nutrient and waste metabolism [126]. In AIS, astrocytes are involved in the acute injury but also function to contain the damaged brain region through formation of a glial scar [127]. Immediately following AIS, the cellular ionic perturbations and subsequent ATP depletion not only affect neurons and glia but also cause swelling of endothelial cells, causing disruption of the tight junctions between them and the connections with astrocytic endfeet, thereby reducing the integrity of the BBB [84]. The activated endothelial cells are known to then stimulate EV release from astrocytes and microglia [128,129]. There is an interesting cross talk between microglia and astrocyte EV secretion. ATP has been shown to stimulate and alter microglia EV release, which in turn triggers astrocytes to take on an inflammatory phenotype [121]. Moreover, ATP released from astrocytes triggers microglial EV release with increased IL-1β cargo [130,131]. Interestingly, a recent study assessing <200 nm EV profiles in the mouse brain during physiologic conditions vs. 24 h post transient MCAO demonstrated microglial EVs predominate at baseline, whereas following ischemia, astrocytic-derived EVs predominate [89]. Interestingly, this was tied with the prion protein PrP being enriched in EVs after 24 h post transient stroke, suggesting a crucial role of PrP in the signaling mechanisms among brain cells after stroke [89]. 

Common protein markers for astrocyte-derived EVs include the astrocyte-specific proteins excitatory amino acid transporter1 (EAAT1/GLAST) and glial fibrillary acidic protein (GFAP) [103]. Astrocytes have been shown to release EVs containing synapsin I, a glycoprotein capable of modulating neurite growth, when cultured with 75–80 mM potassium chloride (KCl) [132]. Potassium elevation serves as an experimental trigger of recoverable SD-like events as seen in migraine aura [15,133,134]. Additionally, astrocytes play a critical role in many of the subcellular events surrounding SD [135]. It remains possible that this finding could translate to the more sinister SD seen in stroke and therefore reflect an upregulated EV constituent tied directly with infarct propagation. Astrocytes are also known to alter EV content following exposure to pro-inflammatory cytokines. Chaudhuri and colleagues demonstrated that treatment with ATP, IL-1β, and TNF-α increases the expression of 7, 10, and 15 distinct miRNAs, respectively, in astrocyte-derived EVs relative to the treatment control [136,137]. Additionally, EVs derived from astrocytes pre-treated with OGD have been demonstrated to reduce neuron apoptosis via the miR-7670-3p/sirtuin 1 (SIRT1) upregulation of autophagy [138]. While research is continuously emerging regarding the effects of astrocyte-derived EVs on ischemic brain injury, further characterization is needed of the EVs released by astrocytes at the time of ischemic injury. Importantly, since astrocytes are uniquely tied to BBB integrity, altered astrocytic EV profiles could serve as indicators for BBB dysfunction. 

### 5.5. Oligodendrocyte-Derived Extracellular Vesicles as Biomarkers in Stroke

Oligodendrocytes are the myelinating cells of the CNS. Common markers for oligodendrocyte-derived EVs include oligodendrocyte-specific proteins, oligodendrocyte myelin glycoprotein (OMG), and myelin basic protein (MBP) [103]. Oligodendrocytes not only play an established role in remyelination post ischemic injury but also have been demonstrated to influence neuronal survival [139]. Like the other cell types discussed, oligodendrocytes also release EVs that can change based on their microenvironment. In an in vitro study examining the effects of oligodendrocyte-derived EVs in an oligodendrocyte/neuron co-culture stressed with OGD, the cells exposed to the oligodendrocyte-derived EVs had a significantly higher metabolic activity compared to control cells. These EVs appeared to contain cargo of antioxidant benefit, including enzymes such as catalase and superoxide dismutase 1 (SOD1) [140]. Oligodendrocytes have also been shown to release EVs in response to glutamatergic signaling and other neuronal signals. These oligodendrocyte-derived EVs have been found to contain a vast number of proteins, including tetraspanins, heat shock proteins, myelin proteins PLP and CNP, Alix, and Tsg101, and RNA [141]. In a recent study, exploring the mRNA content of brain-derived EVs in mice 72 h post MCAO, although the majority of mRNA content was microglial in origin, the EV population with the greatest increase was actually derived from oligodendrocytes [142]. Taken together, these results reveal that oligodendrocytes are likely to be dynamic players in stroke outcome. Overall, knowledge of oligodendrocyte-derived EVs is limited relative to other cell types within the CNS. Broadly, extensive research efforts are needed to characterize oligodendrocyte-derived EVs. Specifically, profiling oligodendrocyte-derived EVs at different time points throughout the course of stroke would help shed light on oligodendrocyte function in both the initial injury and chronic/recovery stages of stroke. 

## 6. The Importance of Cell Origin 

As demonstrated, EVs and their cargo are a promising means of peripherally assessing CNS cell function. Regarding stroke, miRNA represents a more highly studied component of EV cargo. Not mentioned in the above sections, there have been several other non-cell-specific key findings concerning miRNA fluctuations and stroke that have previously been outlined in recent reviews [69,74]. It is important to note that in order to interpret miRNA fluctuations as insights into brain activity post-stroke, cell origin becomes increasingly important. EVs are highly studied in the field of cancer research and many studies have suggested the plausibility of miRNA biomarkers for cancer-specific cellular changes. Pritchard and colleagues, however, demonstrate that blood cells are a major contributor to circulating miRNA levels and therefore suggest caution should be used when interpreting miRNA flux as a cancer-specific marker rather than a blood-cell-based phenomenon [143]. Likewise, as has been recognized by Ji and colleagues, in stroke, it is important to consider other systemic and peripheral organ changes that may impact miRNA flux [144]. 

When thinking about the significance behind differential miRNA expression in stroke, cell origin also plays an important role. For instance, serum EV miRNA-9 and miRNA-124 were found to be significantly higher in AIS compared to controls [144]. While these are highly enriched within the brain, cell origin could still infer important insight into the pathophysiology occurring in the brain. As previously discussed, EV miRNA-124 is known to be released by neurons and is potentially beneficial in excitotoxic events while it is also increased in anti-inflammatory-type microglia-derived EVs [105,123]. MiRNA-9 is also known to function differently in neurons and microglia. In neurons, miRNA-9 is known to play a role in neurogenesis and axonal extension [145,146]. Whereas, in microglia, it has been shown to regulate the cellular activation involved in downregulation of monocyte chemotactic protein-induced protein 1 (MCPIP1). Critically, MCPIP1 plays a key role in controlling the inflammatory response, which suggests that inhibition of miRNA-9 may exert beneficial effects by reducing microglial activation [147]. MiRNA function can also be synergistic in nature, and this should be taken into account when interpreting what an increase in different miRNA may infer about the CNS environment. The small GTP-binding protein Rap2a was found to be a common target of both miRNA-9 and miRNA-124 [148]. Interestingly, Rap2a is only suppressed if both miRNA-9 and miRNA-124 are present, resulting in neural stem cell differentiation and promotion of dendritic branching of differentiated neurons [148]. 

EV miRNAs are also actively involved in pathological changes seen in both stroke and neurodegenerative diseases [149], suggesting that they may be the interlinking factor in the development of PSD. Several publications provide evidence in support of this hypothesis. While plasma serum concentrations of EV miRNA-124 and miRNA-9 are significantly increased acutely post-stroke [144,150,151], miRNA-9 is also found to be upregulated in the serums of AD patients [152,153,154,155]. This suggests that elevated EV miRNA-9 levels post-stroke may be sustained long term, contributing to the emergence of neurodegenerative diseases, but further investigation is required to monitor miRNA-9 changes with stroke resolution. In contrast to miRNA-9, the plasma levels of miRNA-124 are significantly reduced in AD [156,157,158,159,160] and PD [161,162]. Decreased miRNA-124 levels during AD progression lessen its regulation of the beta-secretase enzyme responsible for amyloid generation [159]. This conflicting result stresses that the time elapsed post-stroke is a critical variable when considering how EVs and their cargo reflect cellular function. Indeed, plasma concentrations of miRNA-124 displayed synchronous alterations with stroke progression and resolution [163]. Specifically, miRNA-124 is significantly elevated in the acute injury phase, 1–2 days post-stroke, gradually decreased in the recovery phase up to 30 days post-stroke, and finally restored to normal levels at the late recovery phase by 60 days post-stroke [163]. Given this downward trend, it is possible that miRNA-124 levels may be further decreased below baseline in the long term and facilitate the onset of neurodegenerative diseases [164]. The utility of EV miRNA as clinical biomarkers for post-stroke dementia would require an investigation of a wide panel of miRNAs to increase the specificity and sensitivity of analysis, in addition to characterizing miRNA changes from stroke onset throughout recovery. Additionally, greater insight into the specific cell origin of this EV content would provide more sophisticated insight into the pathophysiologic changes occurring in the brain post-stroke. 

Similar points can also be made for differential EV-contained protein profiles. It is worth noting that the literature surrounding EV cargo post-stroke is heavily focused on the miRNA content and overall investigations of the protein content are relatively scarce. An overall increase in inflammatory proteins in circulating EVs in AIS patients has been found relative to controls. These findings included increased mRNA of TNF, CXCL-, CCL-2, and IL-1β [165]. Additionally, inflammasome proteins in EVs have also been found to be elevated in stroke groups [166]. Collectively, it would be beneficial to determine the origin of these pro-inflammatory EVs, and specifically consider if the increase is seen from peripheral immune cells or is microglial in nature. Additionally, the presence of some proteins from different EV subtypes could indicate entirely different inflammatory profiles within the brain. The presence of MBP, previously noted as a common marker of oligodendrocyte-derived EVs, might be a normal indication of myelin turnover when present in oligodendrocyte EVs [103]. Whereas, if it were found within EVs derived from microglia, one could postulate this may be due to abnormal metabolism of myelin debris. Collectively, more research is needed to characterize cell-specific EV release in distinct acute, subacute, and chronic time periods post-stroke. 

## 7. Conclusions and Future Directions

Stroke represents a prevalent and devastating condition in which significant benefit would come from a means of non-invasively gauging changes in CNS cell function. The characterization of EVs released from the brain represents a novel, peripheral window into the physiologic state of the brain and the cell-specific pathologic changes associated with stroke. While increasing studies are emerging that are examining the content of brain-derived EVs and their therapeutic potential, a vast minority of studies in this field correlate the EV content released during stroke to specific cell types within the CNS. To change this, there needs to be a detailed interrogation of cell-specific EV release and extravasation post-stroke. For instance, even common neuronal EV markers such as L1CAM are not restricted to CNS cells but are also expressed by melanocytes, immune cells, and certain populations of endothelial cells [167].

Many studies have postulated a potential clinical role for EVs in stroke diagnosis and prognosis. For clinical application in acute stroke diagnosis, good EV biomarker candidates must be released in the hyperacute phase, be able to differentiate stroke subtypes, and be easily measurable with simple devices, which highly limits the complexity of targets [74]. Critically, at this point in time, the technology does not exist to perform high-throughput EV analysis in the acute care setting. In contrast, EV biomarkers for stroke prognostics are not restricted by the rapid response time, expanding the complexity and intricacy of target selection. Two potential clinical uses for peripheral EVs post-stroke currently being explored are (1) as prognostic markers of long-term clinical outcome post-stroke and (2) as treatment-monitoring biomarkers in stroke recovery. While chronic EV changes (defined as EVs released 48 h after the acute event) demonstrate early potential promise as predictive biomarkers for stroke progression, follow-up, and treatment monitoring [74], it is important to note that before this conclusion can be drawn, future studies must confirm specificity for stroke over other causes of CNS injury. Such specificity is requisite to validate clinical biomarker potential and is likely to only be elucidated if we first establish a very clear cell-specific understanding of EV release over the course of stroke and other CNS injuries. 

Moving forward, a focus on standardizing isolation and characterization methods along with advances in technology to allow for high-throughput analysis is requisite to one day see clinical application of EV biomarkers. The literature reviewed in this manuscript serves to generate a list of potential candidate EV biomarkers that may infer changes in cellular function in the CNS during stroke. To imply the clinical utility of these candidate biomarkers, future studies must first validate these biomarkers in a clinical setting with the appropriate controls, determine the effect of elapsed time on the biomarker, determine the optimal time for sampling, and finally compare these biomarkers to medical imaging/the current standard of diagnosis/prognosis. However, at this point in time, with currently available technology, we are able to profile the EV content over the course of stroke and relate it to cellular function. In doing so, EVs may serve as a form of a peripheral blood-based window into CNS cellular function. This will enhance our understanding of stroke progression and recovery and could reveal novel targets for future therapeutics. 

EVs themselves may also be used therapeutically for stroke in the future. Mesenchymal stem cells (MSCs) are multipotent stem cells primarily found in bone marrow that have been emerging as a promising means of enhancing post-stroke recovery and are being investigated in pre-clinical stroke models and clinical trials [168]. MSCs are thought to exert their protective effects, including angiogenesis and neurogenesis, via paracrine signaling, at least in part mediated by EVs [169]. Importantly, MSC-derived EV administration is thought to retain the equivalent neuroprotective properties of MSC administration [170]. However, unlike MSCs, MSC-derived EVs are capable of crossing the BBB [171]. Although outside the scope of this review, MSC-derived EVs show early promise as a potential therapeutic for stroke. 

Collectively, a better understanding of the timing by which specific cell types within the CNS release EVs and how their cargo changes over time throughout the course of stroke presents an exciting opportunity to increase our knowledge of changes in cellular function manifesting stroke pathophysiology. A means of peripherally assessing CNS cellular function would allow for clinical sampling at distinct timepoints post-stroke in living stroke patients. Understanding the unique cellular events occurring in the recovering patient could reveal novel targets for future treatments. 

## Data Availability

Not applicable.
